# Natural aging and ovariectomy induces parallel phosphoproteomic alterations in skeletal muscle of female mice

**DOI:** 10.18632/aging.204959

**Published:** 2023-08-14

**Authors:** Mina P. Peyton, Tzu-Yi Yang, LeeAnn Higgins, Todd W. Markowski, Kevin Murray, Cha Vue, Laurie L. Parker, Dawn A. Lowe

**Affiliations:** 1 Department of Rehabilitation Medicine, Division of Rehabilitation Science, University of Minnesota – Twin Cities, Minneapolis, MN 55455, USA; 2 Department of Computer Science, Bioinformatics and Computational Biology Program, University of Minnesota, Minneapolis, MN 55455, USA; 3 Department of Biochemistry, Molecular Biology, and Biophysics, University of Minnesota – Twin Cities, Minneapolis, MN 55455, USA; 4 Department of Rehabilitation Medicine, Division of Physical Therapy, University of Minnesota – Twin Cities, Minneapolis, MN 55455, USA

**Keywords:** estrogen deficiency, CAST, MAPK, PKA, calcineurin

## Abstract

The loss of skeletal muscle strength mid-life in females is associated with the decline of estrogen. Here, we questioned how estrogen deficiency might impact the overall skeletal muscle phosphoproteome after contraction, as force production induces phosphorylation of several muscle proteins. Phosphoproteomic analyses of the tibialis anterior muscle after contraction in two mouse models of estrogen deficiency, ovariectomy (Ovariectomized (Ovx) vs. Sham) and natural aging-induced ovarian senescence (Older Adult (OA) vs. Young Adult (YA)), identified a total of 2,593 and 3,507 phosphopeptides in Ovx/Sham and OA/YA datasets, respectively. Further analysis of estrogen deficiency-associated proteins and phosphosites identified 66 proteins and 21 phosphosites from both datasets. Of these, 4 estrogen deficiency-associated proteins and 4 estrogen deficiency-associated phosphosites were significant and differentially phosphorylated or regulated, respectively. Comparative analyses between Ovx/Sham and OA/YA using Ingenuity Pathway Analysis (IPA) found parallel patterns of inhibition and activation across IPA-defined canonical signaling pathways and physiological functional analysis, which were similarly observed in downstream GO, KEGG, and Reactome pathway overrepresentation analysis pertaining to muscle structural integrity and contraction, including AMPK and calcium signaling. IPA Upstream regulator analysis identified MAPK1 and PRKACA as candidate kinases and calcineurin as a candidate phosphatase sensitive to estrogen. Our findings highlight key molecular signatures and pathways in contracted muscle suggesting that the similarities identified across both datasets could elucidate molecular mechanisms that may contribute to skeletal muscle strength loss due to estrogen deficiency.

## INTRODUCTION

Skeletal muscle is the most abundant tissue in the human body, making up approximately 40% of total body mass in a healthy adult. Muscle is crucial in controlling our movements and posture, protecting internal organs and tissues, storing energy, and regulating body temperature and metabolism. As muscle contracts, the sarcomere, the basic contractile unit in skeletal muscle, shortens and generates molecular force (i.e., muscle strength). The loss of muscle strength significantly impacts the activities and quality of life of the aging population. Age-related muscle strength loss occurs earlier in females than males [1, 2]. Compared to male counterparts, postmenopausal women experience decreased functional capacity, greater strength declines, impairments in muscle repair, and increased sarcopenia and osteoporosis rates with age [3, 4]. Poor muscle strength in postmenopausal women is identified as a strong risk factor for total mortality [5].

Decline in muscle strength mid-life in females associates with the reduction of estrogen, specifically estradiol (E2), the primary circulating estrogen. Reduction in ovarian hormones (E2 and progesterone) is a natural biological process that concludes with menopause, which occurs between the ages of 40–50 in the United States [6]. With the average life expectancy of females being 79.9 years in the United States [7], more than one-third of a woman’s life is now spent in the postmenopausal phase, i.e., in an estrogen-deficient state. In addition, females may also undergo estrogen deficiency due to other events [8–12], thereby, extending this estrogen-deficient state in the life of many women. Clinical and preclinical studies have shown that estrogen deficiency contributes to muscle strength loss in females [13–17] and can be prevented and/or reduced in perimenopausal and postmenopausal women on estrogen-based hormone therapy compared to those not on therapy [18, 19]. Similarly, in rodent studies, skeletal muscle force generation is lower in ovariectomized (Ovx) females and those treated with E2 had muscle force restored to ovarian-intact females [20].

**Figure 1:**
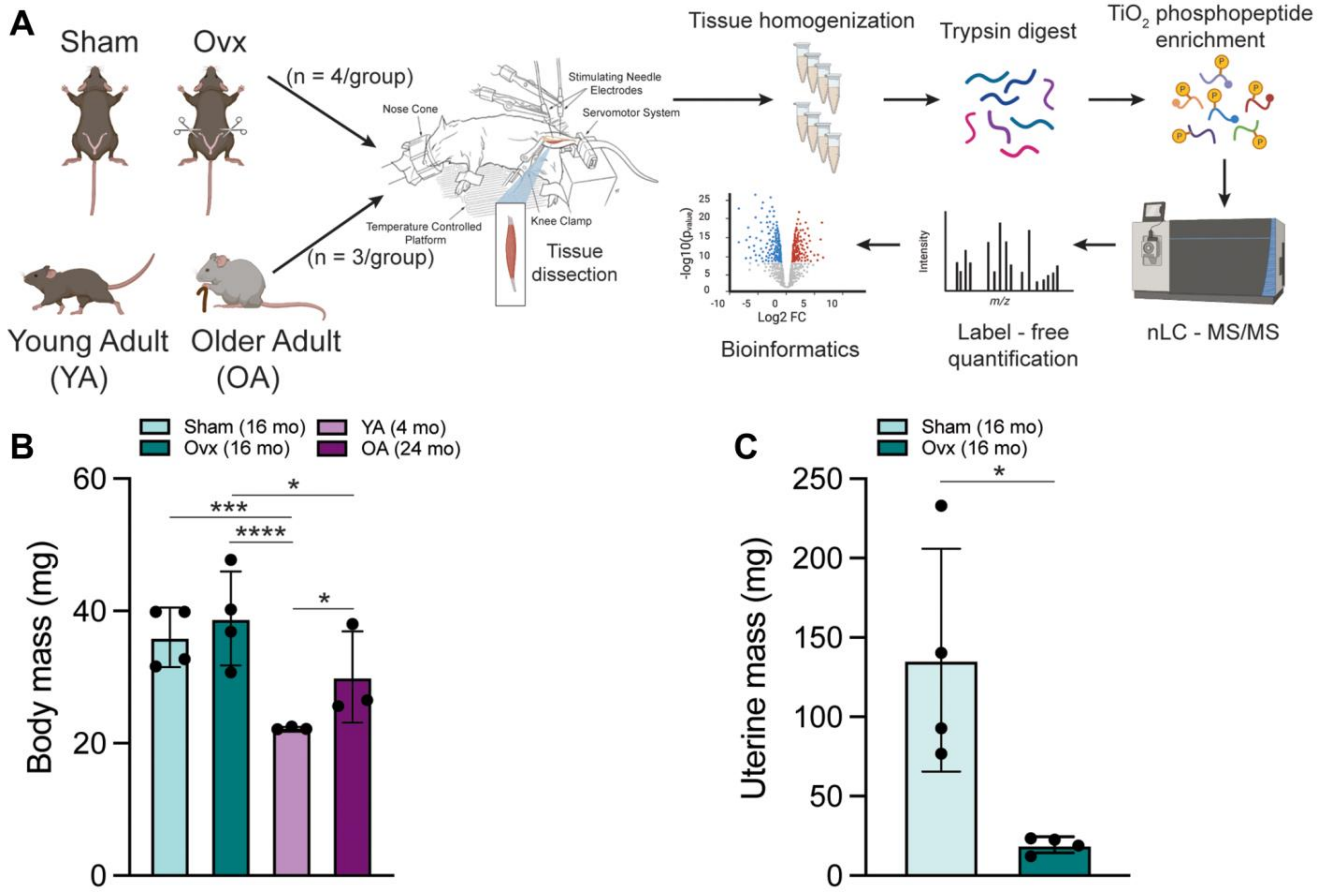
**Schematic of experimental design created with https://www.biorender.com and mouse characteristics.** (**A**) 6 mo C57BL/6J female mice were assigned to a Sham or Ovx group and underwent their respective surgeries. YA and OA mice were 4 mo and 24 mo, respectively. The left leg of anesthetized mice was subjected to *in vivo* contractions and then tibialis anterior muscles were immediately dissected. Frozen TA muscles underwent peptide extraction with trypsin digestion and TiO_2_ phosphopeptide enrichment. nLC-MS/MS was performed on the Orbitrap Fusion Tribrid mass spectrometer for label-free phosphoproteomic analysis. (**B**) Body mass of all four groups of female mice measured before the terminal contraction experiment. Data was analyzed by a one-way ANOVA with Tukey’s multiple comparison test (*p* < 0.001); *n* = 3–4/group. (**C**) Uterine mass of Ovx and Sham mice dissected and weighed after the terminal contraction experiment. Data was analyzed by a pooled *t*-test (Ovx vs. Sham, *p* = 0.016); *n* = 4/group. Values represents mean ± SD. ^*^p < 0.05, ^***^p < 0.001, ^****^p < 0.0001.

Protein phosphorylation, a reversible post-translational modification that fine tunes cellular function and signaling, contributes to and regulates a host of skeletal muscle functions including fiber-type differentiation, muscle hypertrophy, plasticity, regeneration, excitation-contraction coupling, calcium (Ca^2+^) sensitivity, and overall contractile function [21–31]. Previously, estrogen deficiency has been shown to remodel the phosphoproteome of resting, noncontracting muscles [32]. However, skeletal muscle contraction is an external stimulus that induces protein phosphorylation [30, 31]. More, it has been established that phosphorylation of myosin regulatory light chain (pRLC) regulates conformational states of myosin, thus impacting myosin kinetics and binding to actin during contraction, influencing force generation [33]. pRLC has been reported to be 1.8-fold lower in muscle of older compared to young women, with no difference between young and older men [34]. Furthermore, a ~20% decrease in muscle force *in vitro* was correlated with 50% decreased pRLC in Ovx compared to Sham mice [29]. As myosin is only one of many proteins of the sarcomere that undergoes phosphorylation, we hypothesized that estrogen deficiency, via Ovx or natural aging, will modulate phosphorylation of other muscle proteins in response to contraction and force generation. Because skeletal muscle contraction induces protein phosphorylation, [30, 31] this study investigated phosphoproteomic alterations that occur in skeletal muscle immediately after contraction in order to capture estrogen deficiency influences specifically on contraction-induced phosphorylation. We used two mouse models: an ovariectomy model (to represent physiological loss of estrogen) and a natural aging-induced ovarian senescence model (to represent aging more comprehensively, including estrogen loss and other factors), and compared their phosphoproteomic profiles to their respective controls (Ovx vs. Sham and Older Adult (OA) vs. Young Adult (YA); Figure 1A). Performing a comparative analysis across both datasets, the present work identified parallel alterations in contraction-related molecular and cellular signatures, pathways, and upstream regulator activity in skeletal muscle. Importantly, identification of these altered phosphosites and candidate kinases and phosphatases sensitive to the presence of estrogen will help advance our understanding of the contributions of estrogen deficiency to muscle strength loss in aging females.

## RESULTS

### Mouse and Tibialis Anterior (TA) muscle morphometrics and torque analysis

Significant differences in body mass across the groups were measured (*p* < 0.001) (Figure 1B). Mean body masses for Sham, Ovx, YA, and OA mice were 35.8 ± 3.9, 38.4 ± 6.2, 22.3 ± 1.0, and 30.2 ± 5.4, respectively. Uterine mass was significantly different between Ovx and Sham mice (*p* = 0.016), with the mean uterine masses being 19.3 ± 5.1 and 135.7 ± 70.3, respectively (Figure 1C). In addition, cytology confirmed persistent diestrus in Ovx mice and estrous cycling in Sham mice, as expected. Sham and Ovx TA muscle mass were significantly different from OA (*p* = 0.001) (Supplementary Figure 1A), with mean muscle masses of 49.6 ± 4.6, 53.7 ± 3.1, 45 ± 1.2, and 37.1 ± 4.7 mg for Sham, Ovx, YA, and OA, respectively. TA muscle mass normalized to body mass was greater in YA mice than Sham, Ovx, and OA (*p* = 0.003) (Supplementary Figure 1B).

No significant differences among the four groups in absolute torque for pre-tetanic twitch torque, maximal isometric tetanic torque, or post-tetanic twitch torque were measured (0.61 ± 0.09, 2.54 ± 0.53, and 0.84 ± 0.12 mN∙m, respectively for all mice; *p* ≥ 0.158) (Supplementary Figure 1C–1E). However, when body mass was considered, significant differences in normalized torque (torque/body mass) were measured in pre-tetanic twitch torque (*p* = 0.034), tetanic torque (*p* = 0.044), and post-tetanic twitch torque (*p* = 0.003) (Supplementary Figure 1F–1H). These data demonstrate robust muscle contraction intended to trigger protein phosphorylation within each group, as also shown by the twitch torques after the tetanic contraction being greater than the pre-tetanic twitch torques. To note, this study was not designed or powered to detect contractile differences between estrogen-deficient and -replete groups as has been shown previously [15, 29, 35, 36].

### Comparative skeletal muscle phosphoproteomes in Ovx/Sham and OA/YA mice

**Figure 2:**
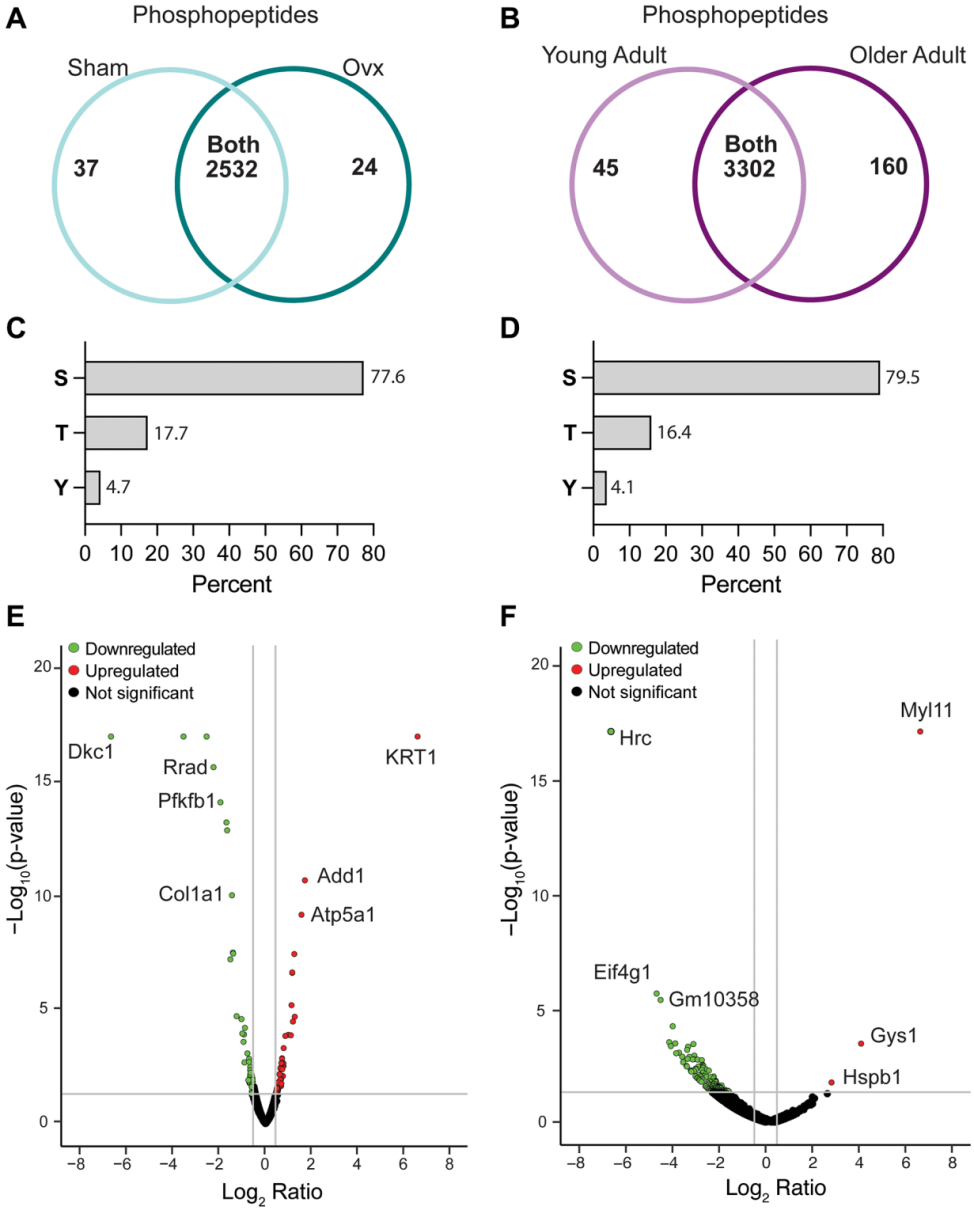
**Characteristics of the Ovx/Sham and OA/YA phosphoproteomes.** Proteome Discoverer (v2.4) was used for database search and identification of phosphopeptides and proteins. Venn diagram of identified phosphopeptides unique to each group and common to both groups in (**A**) Ovx/Sham and (**B**) OA/YA datasets. Prevalence of phosphorylation on amino acid residues serine (S), threonine (T), and tyrosine (Y) in (**C**) Ovx/Sham and (**D**) OA/YA datasets. Volcano plots of differentially regulated phosphopeptides (*p* < 0.05 and 1.4-fold change) in (**E**) Ovx/Sham and (**F**) OA/YA mice.

In the two models of estrogen-deficient mice, a total of 2,593 phosphopeptides and 3,507 phosphopeptides were identified in Ovx/Sham and OA/YA TA muscles, respectively (Figure 2A and 2B). Phosphorylation was most prevalent on serine (Ser, ~ 78%), followed by threonine (Thr, ~ 17%), and tyrosine (Tyr, ~ 4%), which is consistent with literature and was similar across both datasets (Figure 2C and 2D) [37], demonstrating reproducible sample preparation and peptide detection. Further analysis identified 222 and 408 significant and differentially regulated phosphopeptides in Ovx/Sham and OA/YA datasets, respectively (Figure 2E and 2F).

The datasets were processed to identify intersecting proteins and phosphosites, which were then deemed to be associated with estrogen deficiency. After filtering for robustness (i.e., relative abundance detected in at least 3 out of the 4 biological replicates in Ovx or Sham and 2 out of 3 biological replicates in OA or YA groups), 66 estrogen deficiency-associated proteins were identified (Supplementary Table 1). Of these 66 estrogen deficiency-associated proteins, four were significant and differentially phosphorylated (eukaryotic translation initiation factor 4E-binding protein 1, ankyrin repeat domain-containing 2, heat shock protein beta-6, and heterogeneous nuclear ribonucleoprotein U-like protein 2) in the OA/YA Adult dataset. Two (phosphoacetylglucosamine mutase and myc box-dependent interacting protein1) were differentially phosphorylated in the Ovx/Sham dataset.

A total of 21 estrogen deficiency-associated phosphosites were found in the two datasets, of which 4 phosphosites were differentially regulated in both, tumor protein D54 Ser-166, ATP synthase subunit alpha Ser-521, calpastatin (CAST) Ser-82, and H/ACA ribonucleoprotein complex subunit DKC1 (DKC1) Ser-481 (Table 1). CAST Ser-82 and DKC1 Ser-481 were the only two phosphosites that had the same directionally across both datasets, upregulation and downregulation, respectively in estrogen-deficient mice. Although we did identify estrogen deficiency-associated phosphosites on six sarcomeric proteins (myozenin-1 Ser-134, desmin Ser-28, nexilin Ser-559, junctophilin-1 Ser-501, troponin T Ser-2, and myosin regulatory light chain 2 Ser-16), only myozenin-1 Ser-134 was differentially regulated in the OA/YA dataset; whereas, myozenin-1 Ser-164 and troponin T Ser-2 were differentially regulated in the Ovx/Sham datasets. These data show that Ovx and OA mice compared to their respective control, Sham or YA, had some similar phosphoproteomic alterations in contracted muscles induced by estrogen deficiency.

**Table 1:** Estrogen deficiency-associated phosphosites in Ovx/Sham and OA/YA datasets.

**Uniprot accession ID**	**Gene symbol**	**Protein description**	**Phosphosite (Score)**	**Log2 ratio: Ovx/Sham**	**Log2 ratio: OA/YA**
A2AUD5	*Tpd52l2*	Tumor protein D54	S166 (100)	6.64	−6.64
Q03265	*Atp5f1a*	ATP synthase subunit alpha, mitochondrial	S521 (100)	1.61	−6.64
Q8CE04	*Cast*	Calpastatin	S82 (100)	6.64	6.64
Q9CQH3	*Ndufb5*	NADH dehydrogenase [ubiquinone] 1 beta subcomplex subunit 5, mitochondrial	S182 (100)	1.04	−6.64
Q9ESX5	*Dkc1*	H/ACA ribonucleoprotein complex subunit DKC1	S481 (100)	−6.64	−6.64
Q9JK37	*Myoz1*	Myozenin-1	S134 (100)	3.23	6.64
Q5EBG6	*Hspb6*	Heat shock protein beta-6	S16 (100)	−0.80	−3.38
A0A1D5RLD8	*Gm10358*	Glyceraldehyde-3-phosphate dehydrogenase	T182 (100)	−0.58	−2.88
P31001	*Des*	Desmin	S28 (100)	−0.87	−2.53
P48678	*Lmna*	Prelamin-A/C	S390 (100)	−0.93	−2.18
A0A494B9J0	*Ankrd2*	Ankyrin repeat domain-containing protein 2	S321 (100); T325 (100)	−1.09	−2.28
G5E8J6	*Hrc*	Histidine rich calcium binding protein	S104 (100)	−3.25	−1.33
Q9CYR6	*Pgm3*	Phosphoacetylglucosamine mutase	S64 (100)	1.30	−1.51
A0A0G2JEX1	*Nexn*	Nexilin	S559 (100)	3.54	−1.40
A0A0A0MQC7	*Mapt*	Microtubule-associated protein	S188 (100)	−6.64	−0.61
G5E8J6	*Hrc*	Histidine rich calcium binding protein	S354 (100)	0.36	−1.17
P48678	*Lmna*	Prelamin-A/C	S390 (100); S392 (100)	−0.10	−1.19
Q9ET80	*Jph1*	Junctophilin-1	S501 (100)	3.43	1.14
Q9JK37	*Myoz1*	Myozenin-1	S164 (100)	−6.64	−1.40
A0A0R4J1B1	*Tnnt3*	Troponin T	S2 (100)	−6.64	−0.42
P97457	*Mylpf*	Myosin regulatory light chain 2	S16 (99)	0.45	−0.01

The red font denotes significant and differentially regulated phosphosites (BH *p*-value < 0.05 and |FC| ≥ 1.4).

### GO term overrepresentation analysis

**Figure 3:**
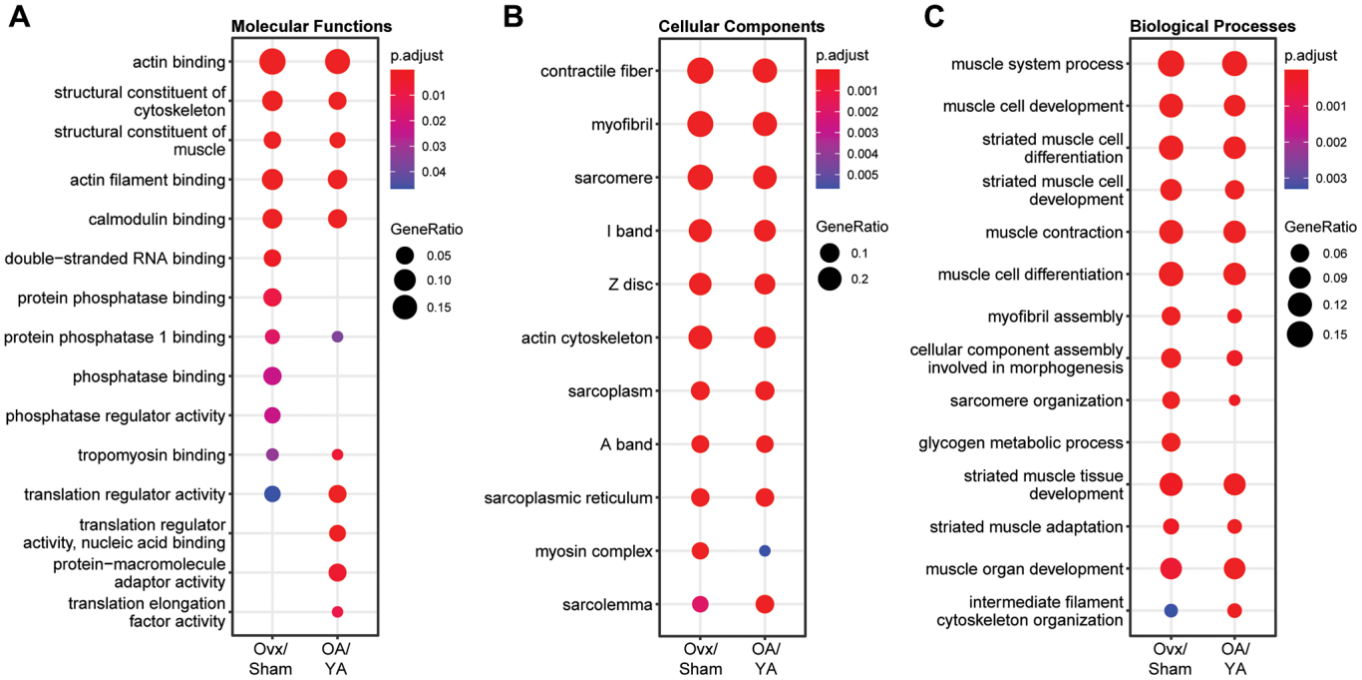
**Comparative GO term enrichment analysis between Ovx/Sham and OA/YA.** Phosphopeptides were mapped back to their precursor protein and submitted for Gene Ontology (GO) overrepresentation analysis using the clusterProfiler package in R. The top 10 overrepresented GO terms in the dataset for (**A**) molecular functions, (**B**) cellular components, and (**C**) biological processes are listed. *P*-value was adjusted using Benjamini – Hochberg post-hoc analysis for multiple comparison. Significant GO terms were accepted at *p*.adjusted < 0.05.

To gain insight on the biological significance of estrogen deficiency in the skeletal muscle phosphoproteome after contraction in Ovx and OA mice, a comparative GO term overrepresentation analysis was performed. The top 10 most overrepresented GO terms for cellular compartment, molecular function, and biological process are shown in Figure 3A–3C, respectively. There was significant overrepresentation of GO terms (FDR < 0.05) across all GO domains with numerous similarities between both datasets. GO molecular function analysis had similar terms enriched in both datasets relating to actin/actin filament binding, structural constituent of the cytoskeleton and muscle, calmodulin binding, tropomyosin binding, and translation regulator activity. However, there was a preferential enrichment in phosphatase activity in the Ovx/Sham dataset; whereas, a preferential enrichment in translation activity in the OA/YA dataset (Figure 3A). All top 10 GO cellular component terms pertaining to the muscle fiber were enriched across both datasets, with reduced enrichment in the myosin complex in the OA/YA dataset. Likewise, all GO biological process terms except one, glycogen metabolic process, was enriched across both datasets. Overall, the top 10 GO term enrichments across many molecular functions, and essentially all cellular components and biological processes were consistent in the Ovx/Sham and OA/YA datasets suggesting that estrogen deficiency confer parallel changes in the phosphoproteome of contracted muscles.

### KEGG and Reactome pathway overrepresentation analysis

**Figure 4:**
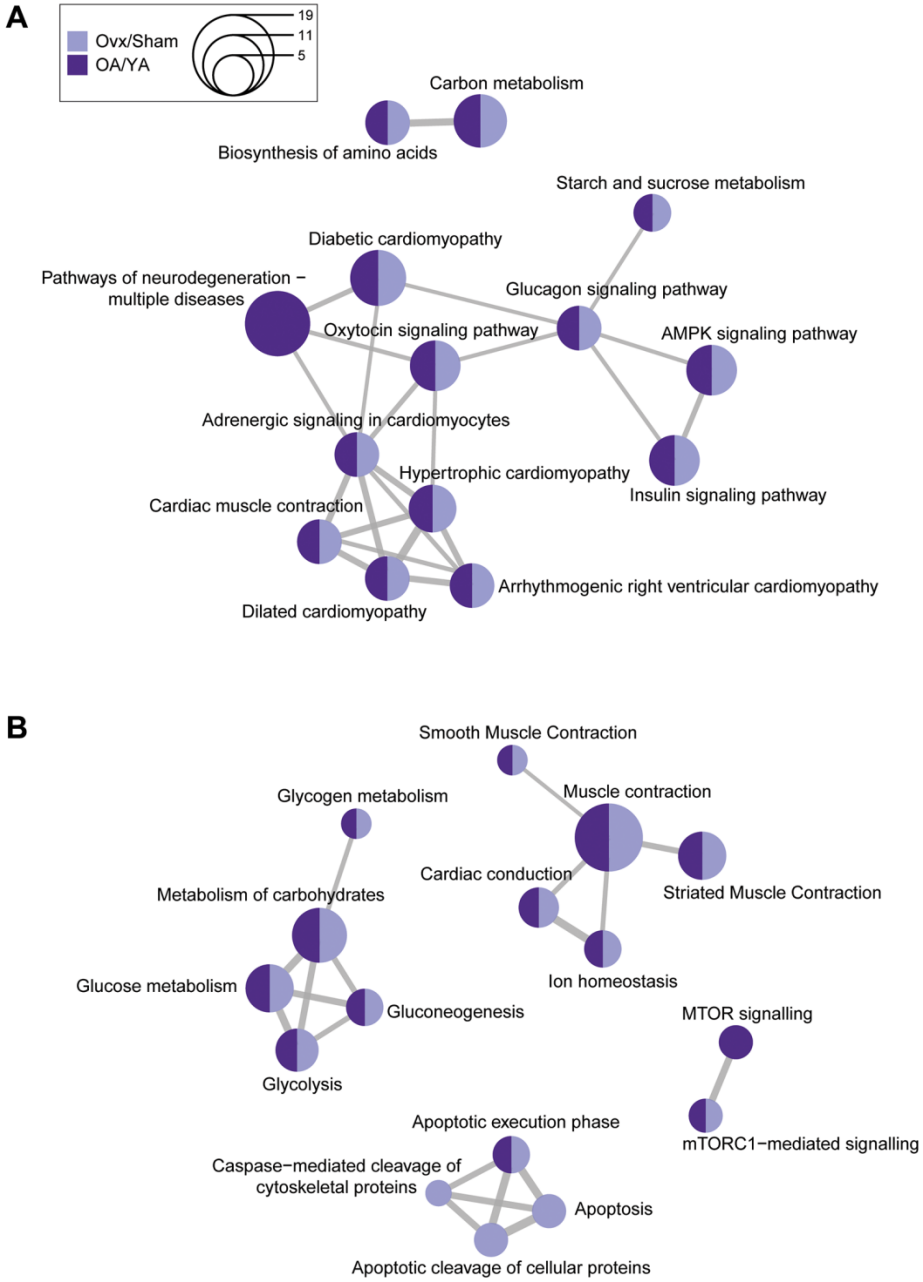
**Comparative KEGG and Reactome pathway enrichment analysis between Ovx/Sham and OA/YA.** Significant and differentially regulated phosphopeptides (adjusted *p*-value < 0.05 and |FC| ≥ 1.4) were mapped back to their precursor protein and the list was submitted to R for analysis of KEGG and Reactome pathways. The top 10 overrepresented (**A**) KEGG and (**B**) Reactome pathways were clustered using K-means clustering. The size of the circle represents the number of proteins associated to the pathway, and the connecting lines represents the strength of the similarity (i.e., shorter and thicker lines correspond to stronger similarity and inversely.

To further explore biological pathways associated with estrogen deficiency in the datasets, overrepresentation analysis of KEGG and Reactome pathways was performed. Using the K-means clustering algorithm, mutual overlapping pathways were clustered together via the similarity between nodes, and the stronger the similarity, the shorter and thicker the connecting lines (Figure 4). Similar to the GO term analyses, among the top 10 pathways, all but one KEGG and three Reactome pathways were associated across both datasets. Only the “pathways of neurodegeneration – multiple diseases” from the KEGG analysis was unique to the OA/YA dataset. Out of the four apoptosis related pathways from the Reactome analysis, three were unique to the Ovx/Sham dataset suggesting that apoptosis was more of a factor for Ovx than it was for natural OA mice. In summary, many enriched KEGG and Reactome pathways were similar across both datasets suggesting that these pathways may be sensitive to estrogen levels and may contribute to muscle contractile dysfunction.

### IPA’s predicted downstream effects and upstream regulator analytics

A comparative analysis between the two core analyses was then performed to identify parallel activation states via IPA’s Z-score algorithm across IPA-derived molecular and cellular functions, canonical pathways, and upstream regulators. For reference, IPA's Z-score indicates a predicted overall activation or inhibition of functions/pathways and activated or inhibited upstream regulators, where a negative Z-score signifies inhibition/inhibited and a positive Z-score signifies activation/activated [38].

#### IPA molecular and cellular functions analysis

**Figure 5:**
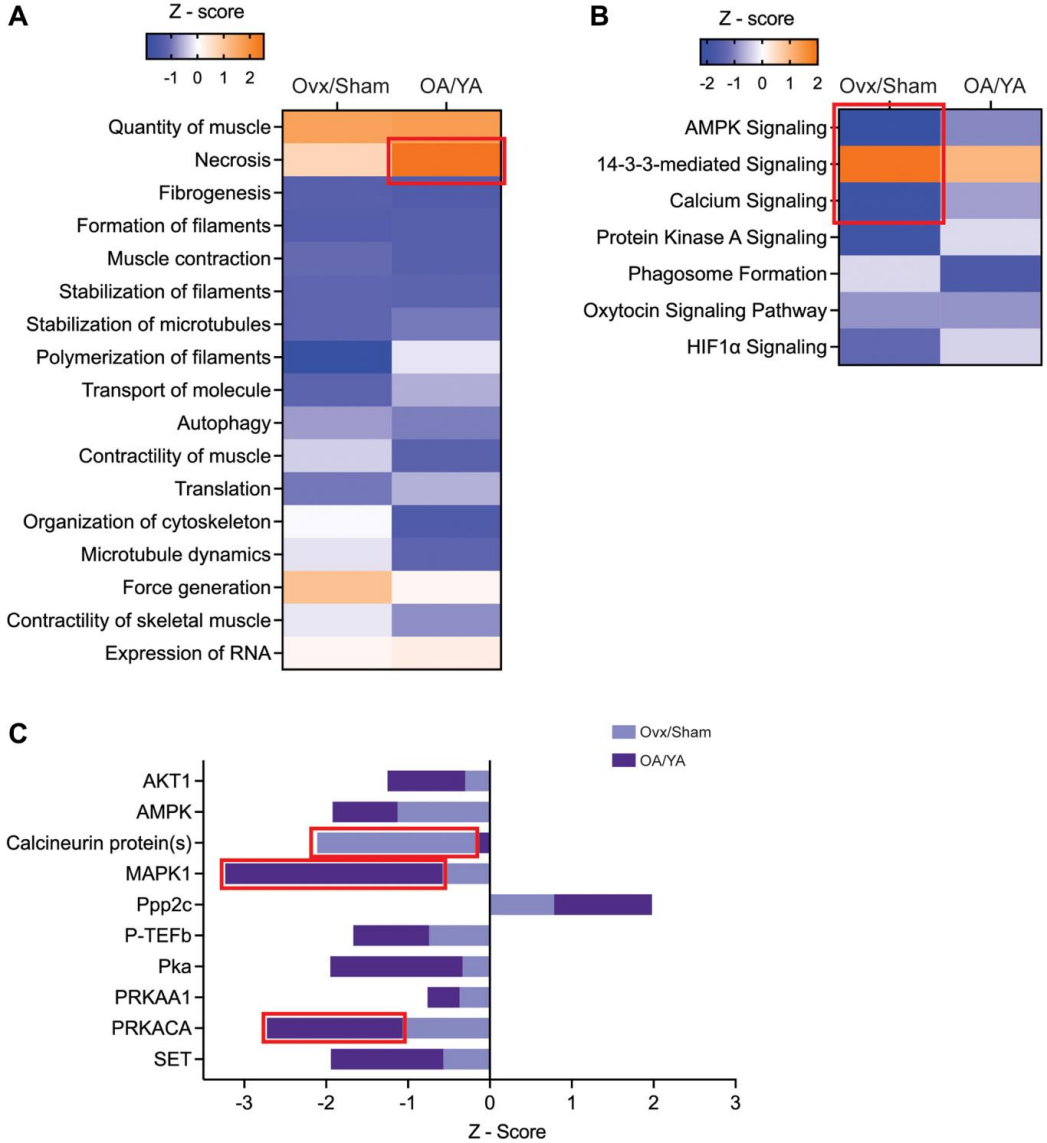
**IPA’s predictive downstream effect and upstream regulator analytics between Ovx/Sham and OA/YA. Phosphopeptides identified in both the Ovx/Sham and OA/YA datasets were submitted to IPA for comparative analysis.** (**A**) canonical pathways, (**B**) functions, and (**C**) kinases and phosphatases using IPA’s predictive activation Z-score to determine downstream and upstream effects of estrogen deficiency. Pathways, functions, and kinases identified in both datasets are represented. Significant Z-scores were accepted at |Z| ≥ 2. Red box denotes significant Z-scores.

Seventeen biological processes were identified across both datasets with similar directionality in activation and were broadly related to muscle quantity and function, filament formation and stabilization, transport of molecules, autophagy, and RNA expression and translation (Figure 5A). Four molecular and cellular functions (quantity of muscle, necrosis, force generation, and expression of RNA) were in activation states, with necrosis activation being significant (Z-score = 2.52) in the OA/YA dataset. The remaining molecular and cellular functions were in inhibition states, with a strong inhibition of polymerization of filaments (Z-score = 1.94) in the Ovx/Sham dataset. These parallel molecular and cellular functions identified across both datasets suggest a compromise not only in the force-generating capacity of skeletal muscle but also mechanisms involved in skeletal muscle maintenance and integrity when estrogen is deficient.

#### IPA canonical pathway analysis

Seven canonical pathways were identified having the same directionality of activation across both datasets, with all showing inhibition except for 14-3-3 protein mediated signaling which was activated in an estrogen deficient condition (Figure 5B). All pathways were significantly enriched (*p* < 0.05) across both datasets except for phagosome formation in the OA/YA dataset (*p* = 0.299). The top three canonical pathways, AMPK signaling, 14-3-3 protein mediated signaling, and calcium signaling, had significant Z-scores in the Ovx/Sham dataset (-2.23, 2.0, and -2.0, respectively). The similarities in inhibition or activation of canonical pathway profiles indicate important calcium, kinase, and cellular signaling in skeletal muscle that may be compromised in estrogen-deficient mice, which could in turn contribute to changes in muscle force generation.

#### IPA upstream regulatory analysis

Ten upstream regulators (i.e., candidate kinases and phosphatases) were identified across both datasets (Figure 5C). All candidate kinases and phosphatases were inhibited (negative Z-scores) with the exception of serine/threonine-protein phosphatase 2A catalytic subunit (PPP2C) being activated in both the Ovx/Sham (Z-score = 0.79) and OA/YA (Z-score = 1.98) datasets. Mitogen activated protein kinase 1 (MAPK1 also known as ERK2, Z-score = −3.23) and cAMP-dependent protein kinase (PKA) catalytic subunit alpha (PRKACA, Z-score = −2.74) were significantly inhibited and SET (Z-score = −1.94) and AMPK (Z-score = −1.92) was highly inhibited in the OA/YA compared to the Ovx/Sham dataset (Z-scores = −0.58, −1.07, −0.57, and −1.13 for MAPK1, PRKACA, SET, and AMPK, respectively). Calcineurin proteins(s) (Z-score = −2.11) was the only phosphatase regulator that was significantly inhibited in the Ovx/Sham dataset compared to the OA/YA (Z-score = −0.13). Overall, the parallel activation status of these candidate kinases and phosphatases across both datasets suggests sensitivity to the loss of estrogen in skeletal muscle of female mice, with more robust inhibited or activated score in the OA/YA group potentially due to the compounded effects of aging.

## DISCUSSION

The purpose of this study was to utilize two models of female estrogen deficiency, ovariectomy and natural aging-induced ovarian senescence, to identify estrogen sensitive alterations in the phosphoproteomic landscape of skeletal muscle after contraction. We identified parallel alterations in molecular and cellular signatures and pathways in both models related to skeletal muscle contractile function and structural integrity coupled with enriched pathways for AMPK, 14-3-3, and calcium signaling. This work provides insight into phosphorylation alterations and potential candidate kinases and phosphatases related to force-generating capacity of skeletal muscle affected by estrogen deficiency that may contribute to muscle strength loss in females.

Estrogen deficiency by Ovx influences phosphorylation of muscle proteins in resting, noncontracted TA muscles; including proteins related to calcium handling and identifying AMPK as a candidate kinase [32]. Not surprisingly, here in response to contraction, phosphorylation alterations in the TA muscle from two models of estrogen deficiency, Ovx and aged, ovarian senescent mice, were measured and inhibition of AMPK and calcium signaling pathways were identified as well. Dysregulation in AMPK signaling and perturbed calcium handling in skeletal muscle is consistent with other reports in Ovx and aging mice [39–41]. Taken together, these results suggest that whether in resting, noncontracted or contracted skeletal muscle, estrogen deficiency may modulate phosphorylation alterations pertaining to AMPK signaling and calcium handling in females that may impact force generation.

In addition, calcium-mediated proteolysis appears to be affected by the estrogenic environment. Significant upregulation of CAST (a calpain inhibitor) Ser-82 phosphorylation combined with altered phosphorylation of calpain substrates - downregulation of desmin and troponin T in both datasets and downregulation of myosin regulatory light chain phosphorylation in OA/YA dataset with upregulation in the Ovx/Sham dataset - imply abrogation of calpain activities in contracted muscle. Hyperphosphorylation of CAST positively regulates calpain inhibition [42], and phospho-modifications on calpain substrates has been shown to regulate the susceptibility of the substrate to calpain degradation [43]. Calpain-mediated proteolytic activities in skeletal muscle are crucial for myofibrillar protein turnover and aid in muscle plasticity through disassembly of the myofibril [44]. Impaired proteostasis by decreased proteolytic activity and/or increased protein aggregation – a hallmark in skeletal muscle aging [45], may contribute to the loss of strength in females due to estrogen deficiency. Interestingly, such estrogen-sensitive phosphorylation alterations in calpain-related proteolytic activities appear to be mediated by muscle contraction, as CAST was not significantly upregulated in noncontracted Ovx female muscle [32].

The present study suggests that loss of estrogen modulates kinase and phosphatase activities in contracted muscle that may affect the force-generating capacity in female mice, as predicted by the significant changes in activation status from IPA’s upstream regulator analysis. All identified kinases and phosphatases had analogous directionality of activation in both models of estrogen deficiency with MAPK1/ERK2 and PRKACA predicted to be significantly inhibited in the OA/YA Adult dataset. Identification of the involvement of the MAPK pathway via MAPK1/ERK2 as a potential candidate kinase sensitive to estrogen level is consistent with previous work in contracted *ex vivo* and noncontracted muscle [29, 32]. Total MAPK1/ERK2 levels have also been reported to be significantly decreased in skeletal muscle from Ovx compared to Sham mice after a fatiguing contraction protocol [39], and reduced activation was observed after a bout of resistance exercise in older men compared to young men [46]. Furthermore, C2C12 cells exposed to hydrogen peroxide exhibited cytoskeleton disorganization, mitochondrial redistribution, and fragmented nuclei – features associated with apoptosis, and were rescued upon E2 pretreatment, which was found to exert anti-apoptotic effects via ERK and p38 MAPK activation [47]. Therefore, downregulation of MAPK1/ERK2 activity in contracted muscle may indicate increased activation in apoptotic pathways and increased perturbations of the cytoskeleton, consistent with our enrichment analyses.

Another candidate kinase identified from IPA’s upstream regulator analysis was PRKACA. PRKACA is the catalytic subunit alpha of PKA that is released upon binding of cAMP to the regulatory subunit dimer of PKA. Unlike MAPK1/ERK2, there is limited information on the specific role of PRKACA in skeletal muscle. However, PKA has also been identified as a potential candidate kinase sensitive to E2 [29]. Overexpression of the PKA catalytic domain in skeletal muscle was revealed to inhibit Forkhead box O activity and contribute to muscle remodeling [23]. In addition, incubation of skeletal muscle single fibers with PKA enhanced contractile function by modifying protein-binding protein C in older compared to young men [48]. Importantly, it is well established that PKA regulates calcineurin [49], which was also predicted to be downregulated in contracted muscle from both estrogen deficient models. Calcineurin inhibition has been shown to induce muscle defects, such as fiber atrophy, immature myotube formation, calcification, and inflammation [50]. The impact of PKA and/or calcineurin have been extensively studied and details on their impact on hypertrophy, regeneration, metabolism, and muscle disorders in skeletal muscle can be found in a number of exceptional reviews [51–56]. The implication of both PKA and calcineurin being inhibited in our two datasets suggest comprised contractile function, fiber maturation, and muscle remodeling that may in turn contribute to decreased force-generating capacity of skeletal muscle in estrogen-deficient females, whether via ovariectomy or natural aging-induced ovarian-senescence.

The current study examined the impact of estrogen deficiency in the skeletal muscle phosphoproteome after contraction and force generation and identified corresponding alterations in protein phosphorylation, pathways, and upstream regulators in both Ovx and natural aged, ovarian-senescent female mice. The results provide two rich datasets for further study of estrogen deficiency-induced phosphoproteomic alterations in female skeletal muscle. For example, further analyses could probe estrogen sensitivity changes of the phosphoproteomic landscape in the transition from resting muscle to contracting muscle. Our study on noncontracted muscle [32] and here on contracted muscle utilized the tibialis anterior muscle of the leg, which in mice consists of fibers expressing type 2 myosin heavy chain. Thus, future studies comparing estrogen deficiency-induced phosphoproteomic alterations in muscles composed of type 1 fibers to datasets from TA muscle would be highly informative. Furthermore, to confirm estrogen deficiency was the unifying factor in the surgical and aging estrogen-deficiency models used here, an estradiol replacement intervention in either or both of the models would be ideal. In summary, our results from contracted skeletal muscle highlight CAST Ser-82 as a candidate phosphosite, and MAPK1/ERK2, PRKACA, and calcineurin as candidate upstream regulators sensitive to estrogen deficiency that may contribute to changes in the force-generating capacity of skeletal muscle. Future studies based on these bioinformatic and computational modeling analyses will be key confirmatory experiments to test the extent to which the predicted candidate phosphosites, kinases and phosphatases, and pathways affect muscle strength in aging females.

## MATERIALS AND METHODS

### Animals

Female C57BL/6J (6, 4, and 24 mo) mice were purchased from Jackson Laboratories (Bar Harbor, ME, USA). All mice were housed in groups of four to five with access to phytoestrogen-free food and water ad libitum. The room was maintained on a 14:10 h light/dark cycle.

### Experimental design

The study design to investigate the skeletal muscle phosphoproteome after contraction in two model of estrogen deficiency, Ovx vs. Sham (6 mo; *n* = 4/group) and OA (24 mo) vs. YA (4 mo; *n* = 3/group), is summarized in Figure 1A. C57BL/6J female mice were randomly assigned to a sham or ovariectomy surgery. Sham and Ovx mice were 14 mo at the terminal experiment and Ovx mice were estrogen deficient for 32 wk. Vaginal cytology was performed 4 weeks post-surgery and uterine mass was measured at the terminal experiment. OA and YA mice were purchased from Jackson Lab and were acclimatized for two weeks before the terminal experiment. We chose 24 mo of age for the OA group, as C57BL/6J mice are ovarian-senescent as previously determined by cessation of estrous cycling between 13-16 mo [57] and in another study at <20 mo as determined by vaginal cytology and plasma E2 [58]. Age, evaluation of vaginal cytology, and measurement of uterine mass are proxies to indicate estrogen deficiency; a shortcoming of this study is that serum E2 was not measured.

An *in vivo* muscle contraction protocol of the anterior crural muscles was performed on each mouse in all four groups. The protocol (detailed below) primarily consisted of one maximal isometric tetanic contraction. A single tetanic contraction was chosen to mitigate potential complications of muscle fatigue that might occur with multiple maximal contractions. Immediately following the muscle contraction protocol, the contracted tibialis anterior (TA) muscles from anesthetized mice were dissected within 5 min, flash frozen in liquid nitrogen, and stored at −80ºC until sample preparation and phosphopeptide enrichment for nanoflow LC-MS/MS acquisition. The TA muscle was selected for study for several reasons including; (1) consistency with phosphoproteomic results from our previous work on non-contracted muscles [32], (2) specificity of stimulating contraction and measuring peak tetanic and twitch torques of the anterior crural muscles [59], and (3) the muscle is composed of type II fibers [60] rendering it more sensitive to contraction-induced contractile protein phosphorylation relative to a muscle composed of type 1 fibers [61].

### Sham and ovariectomy surgeries

Anesthetized (1.75% isoflurane and 200 ml O_2_ per min) mice received a subcutaneous injection of slow-release buprenorphine (1 mg/kg) immediately prior to surgeries. Bilateral ovariectomy via abdominal incisions were made to locate the ovaries which were excised in Ovx mice, and located but not removed in Sham mice. The abdominal muscle wall incisions were closed with 6–0 silk sutures and 7 mm wound clips closed the skin incision.

### *In vivo* muscle contraction protocol

Mice were anesthetized (1.25% isoflurane and 125 O_2_ per min) and positioned on a temperature-controlled platform as previously described [62]. Briefly, the left knee was immobilized and the left foot secured to an aluminum “shoe” that was attached to the shaft of an Aurora Scientific 300B servomotor. Sterilized platinum needle electrodes (FE212, Grass Technologies, Warwick, RI, USA) were inserted to stimulate the left common peroneal nerve, and voltage and electrode placement were optimized with 3–5 twitch contractions (0.1 ms pulse). Following optimization, an isometric tetanic contraction of the anterior crural muscles (the TA being the primary muscle in that group) was elicited (1000 Hz for 1000 ms with 0.1 ms pulses), with a twitch contraction performed 10 s prior and two post-tetanic twitch contractions at 2 s and 30 s after the tetanic contraction [36, 59].

### Protein extraction, digestion, and phosphopeptide enrichment

Frozen dissected TA muscles were prepared as previously described [32]. Briefly, TA muscles were pulverized into powder with a cryo-grinder (liquid nitrogen cooled mortar and pestle), lysed (10 μl lysis buffer per mg of tissue) in protein lysis buffer (7 M urea, 2 M thiourea, 0.4 M Tris pH7.5, 20% acetonitrile, 4 mM TCEP) with 1X HALT Protease and Phosphatase Inhibitor Cocktail (Thermo Fisher Scientific, Rockford, IL, USA), and sonicated for 5 s using a probe sonicator (Branson Digital Sonifier, Emerson, St. Louis, MO, USA) set at 30% amplitude, all done on ice. After sonication, a 160 μl aliquot of each lysate was placed in the Barocycler^®^ NEP2320 (Pressure Biosciences, South Easton, MA, USA) at 37ºC, with pressure cycles set at 35,000 psi for 20 s, then 0 psi for 10 s for 60 cycles for further protein homogenization. Once pressure cycling was complete, samples were transferred to a new 1.5 ml Eppendorf protein LoBind tube and 200 mM chloroacetamide stock solution was added for a final concentration of 8 mM to alkylate proteins (1:24 dilution) and incubated for 15 min at room temperature. Samples were spun down at 15,000 × *g* for 10 min at 18°C. Aliquots of supernatant were used to determine protein concentration using the Bradford assay. For trypsin digestion, 500 μg total protein was digested with 12.5 ug sequencing grade modified trypsin (Promega, Madison, WI, USA) and incubated at 37°C in a warm air incubator overnight (~ 16 h). Samples were acidified to 0.2% trifluoroacetic acid (TFA) to a final volume of 1 ml and extracted using 1 cc Oasis HLB Solid Phase Extraction cartridges (Waters Corporation, Milford, MA, USA) for clean-up. Briefly, 1cc HLB cartridges were equilibrated by passing 1 mL of 80% acetonitrile, 0.1% TFA over the cartridge followed by passing 1 mL of 0.1% TFA. The sample was passed over the cartridge followed by washing the sample with 1 mL 1% acetonitrile, 0.1% TFA over the cartridge. Peptides were eluted with 0.5 mL 50% acetonitrile, 0.1% trifluoroacetic acid and vacuum dried to remove acetonitrile. Lysates were stored at −80°C until phosphopeptide enrichment. Phosphopeptide enrichment was performed with the High-Select^™^TiO_2_ Phosphopeptide Enrichment Kit (Thermo Fisher Scientific, Rockford, IL, USA). Eluted peptides were dehydrated using a speed-vac.

### Nanoflow LC-MS/MS

Approximately 600 ng of peptide mixture, contained in a 1 μl aliquot of 98:2, water:acetonitrile, 0.1% formic acid, were analyzed in data dependent acquisition mode by liquid chromatography (LC)-nano ESI-mass spectrometry (MS) with an Ultimate 3000 Dionex RSLC nano LC system online with an Orbitrap Fusion Tribrid mass spectrometer (Thermo Fisher Scientific, Rockford, IL, USA). Peptides were separated during a linear gradient with the following profile: 5–22% solvent B in 70 min, 22–35% solvent B over 35 min, and 90% solvent B held for 10 min. Solvent A was water with 0.1% formic acid and solvent B was 80% acetonitrile with 0.1% formic acid. The 50 cm column was packed in house using ReproSil-Pur 120 C18-AQ 1.9 μm (Dr. Maisch, Ammerbuch-Entringen, Germany) in a PicoTip 75 μm inner diameter (New Objective, Littleton, MA, USA). Data acquisition was acquired with the following MS parameters: ESI voltage 2.1 kV, ion transfer tube 275°C; Easy-IC internal calibration; Orbitrap MS1 scan 120k resolution in profile mode from 380–1580 *m/z* with 100 msec injection time; 100% (4 × 10E5) automatic gain control (AGC); MS2 was triggered on precursors with 2–6 charges above 5 × 10E4 counts; MIPS (monoisotopic peak determination) was set to peptide; MS2 settings were: 1.6 Da quadrupole isolation window, higher energy collisional dissociation activation at 35% collision energy, Orbitrap detection with 60K resolution at 200 *m/z*, first mass fixed at 110 *m/z*, 150 msec max injection time, 100% (5 × 10E4) AGC and 40 sec dynamic exclusion duration with +/− 10 ppm mass tolerance.

### Phosphoproteomics database search, phosphoprotein and phosphopeptide quantification

The raw MS files were processed by Proteome Discoverer v2.4 (Thermo Fisher Scientific, Rockford, IL, USA). MS/MS spectra were searched against the UniProtKB/Swiss-Prot *mus musculus* database (55,474 entries, UniProt UP000000589, downloaded November 2019) with the Sequest HT search engine embedded in Proteome Discoverer v2.4. Parameters were set as follows: MS1 tolerance of 15 ppm, MS/MS mass tolerance of 0.05 Da, trypsin (full) digestion with a maximum of two missed cleavages, minimum peptide length of 6 and maximum of 144 amino acids. Cysteine carbamidomethylation (57.02 Da) was set as a fixed modification, and methionine oxidation (15.99 Da), asparagine and glutamine deamidation (0.98 Da), acetylation of the N-terminus (42.01 Da), and phosphorylation of tyrosine, serine, and threonine (79.97 Da) were set as dynamic modifications. A false discovery rate (FDR) of 1% was set for peptide-to-spectrum matches using the Percolator algorithm (v3.02.1) and for protein assignment. Phospho-localization scoring was performed with the IMP-ptmRS v2.0 node and only phosphopeptides with a localization score >0.8 were used for quantification. Unique and razor peptides were used for quantification. Precursor abundance quantification was based on area and normalized by total peptide amount. All peptides were used for normalization for protein quantification; however, only phosphorylated peptides were used for pairwise ratios and protein roll-up.

Label-free quantitation (LFQ) of phosphopeptides were performed with normalized abundances using the Proteome Discoverer LFQ algorithms. The protein ratio was calculated as the geometric median of the phosphopeptide group ratios, and the phosphopeptide group ratios were calculated as the geometric median of all combinations of phosphopeptide ratios from all the biological replicates in the study. Phosphopeptides that had a high 1% FDR confidence were used for further analysis. We applied a maximum *p*-value filter of 0.05 and a minimum relative fold change of phosphoprotein and phosphopeptide expression at 1.4 to the Proteome Discoverer quantification results. Phosphoproteins and phosphopeptides were considered significant and differentially regulated if they had an adjusted *p*-value < 0.05 and were defined as downregulated if they had a fold change ≤ −1.4 or upregulated if they had a fold change ≥ 1.4.

### Kyoto Encyclopedia of Genes and Genomes (KEGG), Reactome, and Gene Ontology (GO) enrichment analysis

Significant and differentially regulated phosphopeptides were mapped back to their precursor protein and the list of proteins was used for overrepresentation analysis using the clusterprofiler and ReactomePA packages in R v4.1.1 [63–65]. Comparative enrichment analysis of the Ovx/Sham and OA/YA datasets were performed for GO terms in all three domains: cellular component, molecular function, and biological process. KEGG and Reactome pathway analyses were performed to identify overrepresented pathways. Cluster analysis using the K-means algorithm was performed on the top 10 KEGG and Reactome pathways. Overrepresented pathways and GO annotation terms were considered significant if they had a Benjamini – Hochberg adjusted *p*-value < 0.05.

### Ingenuity pathway analysis (IPA)

IPA (Qiagen, Redwood City, CA, USA) was used to perform core analyses on the on the phosphopeptides from Ovx/Sham and OA/YA datasets. A comparative analysis across both core analyses was performed in IPA using the activation Z-score algorithm to predict the activation states of pathways, functions, and upstream regulators from both datasets. The Z-score measures how closely the observed expression pattern of the molecules from the datasets compare to the expected expression pattern based on the literature for a particular annotation. Molecules from the dataset that met the cutoffs, |FC| ≥ 1.4 and adjusted *p*-value < 0.05 were considered for analysis.

### Statistical analyses

Relative phosphoprotein quantification was analyzed in Proteome Discoverer v2.4 (Thermo Fisher Scientific, Rockford, IL, USA) using a background *t*-test. Fisher’s exact test was used in IPA to calculate *p*-values for the association or overlap between the identified molecules in the dataset and a given pathway/process/function. Benjamini-Hochberg post-hoc analysis was used to correct for multiple comparisons. The predicted activation state in the pathways/functions/upstream regulators in IPA were measured as a Z-score. Significant activation or inhibition was accepted at |Z| ≥ 2. Significance was accepted at α < 0.05 level.

### Supporting information

Supplementary Materials and Methods and Figures can be found online.

The mass spectrometry proteomics data have been deposited to the ProteomeXchange Consortium via PRIDE [66] partner repository with the dataset identifier PXD035171 (http://www.ebi.ac.uk/pride/archive/projects/PXD035107).

## Supplementary Materials

Supplementary Figure 1

Supplementary Table 1
