# Supplementary material for: Natural aging and ovariectomy induces parallel phosphoproteomic alterations in skeletal muscle of female mice
Source: Aging (Albany NY). 2023 Aug 14;15(15):7362–80. doi: 10.18632/aging.204959 (PMC10457050; doi:10.18632/aging.204959)
Supplement: Supplementary Figure 1 [file aging-15-204959-s001.pdf]

SUPPLEMENTARY FIGURE

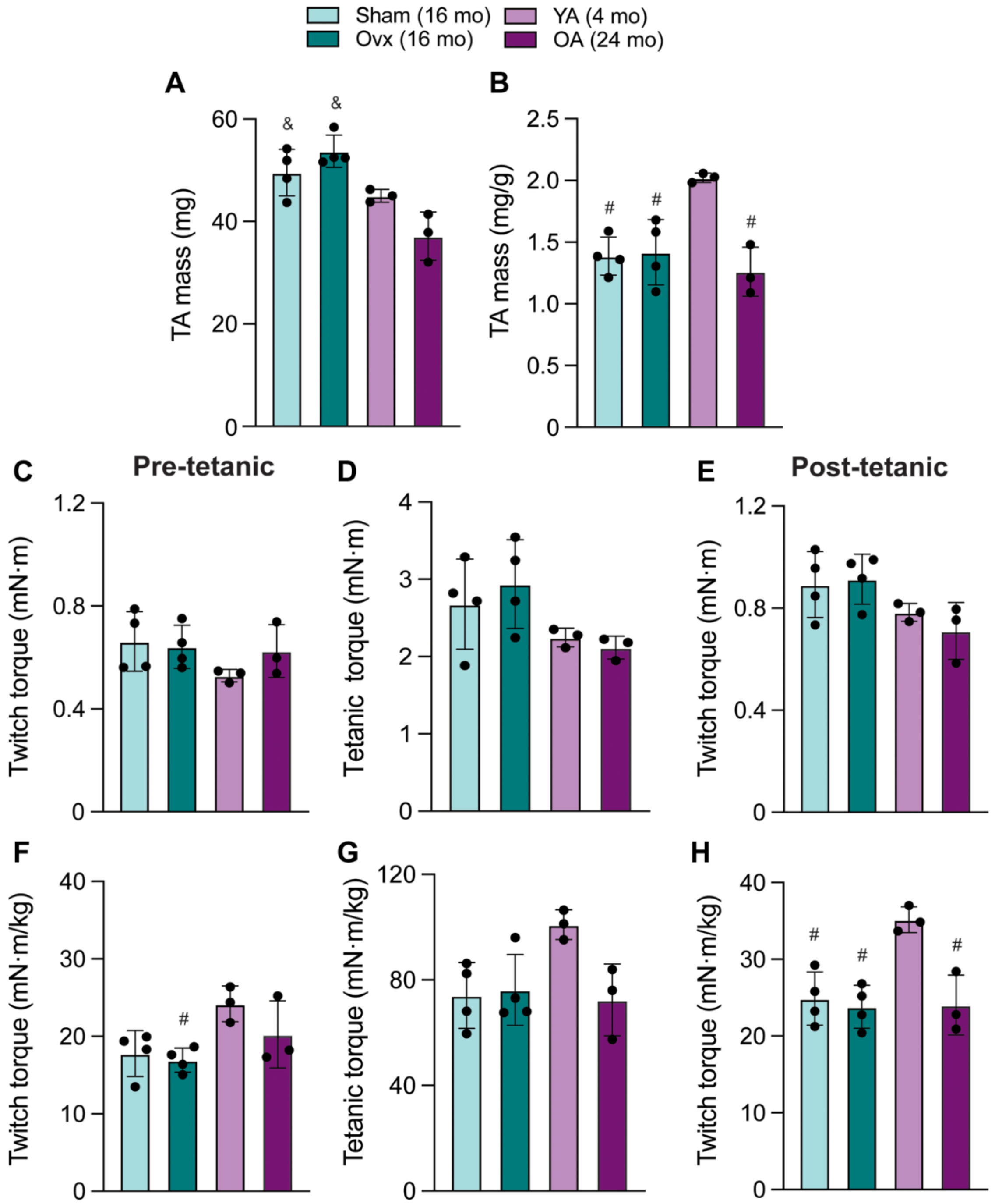

Supplementary Figure 1. TA muscle mass and torque of adult Sham and Ovx mice and Young adult (YA) and Older adult (OA) mice. (**A**) Left TA muscles were immediately dissected after the terminal contraction experiment and weighed. (**B**) TA mass was normalized to mouse body mass. Absolute torque measurements were measured in each mouse (**C**–**E**) and torque measurements were normalized to body mass (**F**–**H**). (**C**, **F**) pre-tetanic twitch torque, (**D**, **G**) maximal isometric tetanic torque, and (**E**, **F**) post-tetanic twitch torque measurements. All data A-H were analyzed by a one-way ANOVA with Tukey's multiple comparison test: significant differences were found in TA mass (p = 0.001), normalized TA mass to body mass (p = 0.003), and normalized torque ($p \leq 0.044$), but no significant differences were found across groups for absolute torque measurements ($p \geq 0.158$); n = 3–4/group). Values represents mean ± SD. & significantly different from OA, # significantly different from YA.
