# Supplementary material for: Natural aging and ovariectomy induces parallel phosphoproteomic alterations in skeletal muscle of female mice
Source: Aging (Albany NY). 2023 Aug 14;15(15):7362–80. doi: 10.18632/aging.204959 (PMC10457050; doi:10.18632/aging.204959)
Supplement: Supplementary Table 1 [file aging-15-204959-s002.docx]

**Supplemental Table 1. Estrogen deficiency-associated phosphoproteins identified in both Ovx/Sham and OA/YA datasets.**

| **Uniprot ID** | **Gene Symbol** | **Protein description** | **Phosphosite (Score): Ovx/Sham** | **Phosphosite (Score): OA/YA** | **Log2 ratio: Ovx/Sham** | **Log2 ratio: OA/YA** |
| --- | --- | --- | --- | --- | --- | --- |
| Q60876 | *Eif4ebp1* | Eukaryotic translation initiation factor 4E-binding protein 1 | T40(88.2); T45(90.8); T69(100) | T36(94.4); T45(99.8) | 0.58 | -2.93 |
| A0A494B9J0 | *NA* | Ankyrin repeat domain-containing protein 2 | S72(99.4); S321(100); T325(100) | S56(100); S72(99.2); S321(100); T325(98.2) | -0.23 | -2.4 |
| Q5EBG6 | *Hspb6* | Heat shock protein beta-6 | S16(100); S157(100) | S16(100) | -0.52 | -2.24 |
| Q00PI9 | *Hnrnpul2* | Heterogeneous nuclear ribonucleoprotein U-like protein 2 | S159(100); S226(98.4) | S159(100); S183(100); S186(100); S191(100); S226(94.1) | 0.71 | 0.71 |
| G3UW82 | *Myh2* | Myosin heavy chain 2, MCG140437 | T257(100); T258(100); T381(100); Y413(100); T415(100); T444(100); S649(99); T667(100); T687(100); S745(100); T761(100); T793(100); T918(100); T986(100); T1000(100); T1026(100); T1028(100); S1044(100); S1095(100); S1135(100); S1147(100); S1165(100); T1198(100); S1206(100); S1240(100); S1268(100); T1282(99.3); S1306(100); S1309(100); T1316(100); S1373(100); Y1382(100); T1390(100); Y1467(99.3); S1483(100); S1498(100); T1504(100); T1520(100); S1577(100); S1603(99.3); T1607(100); S1614(100); T1653(100); S1717(100); T1725(100); T1733(100); T1739(100); S1742(100); | T218(100); S225(100); T257(99.4); T258(99.2); T381(100); Y413(100); T415(100); T444(100); T619(99); S650(98.9); T667(100); S735(100); S745(100); T761(100); T793(100); T944(100); T967(100); T995(100); T1000(100); T1026(100); S1044(100); S1095(100); S1135(100); S1147(100); S1165(100); T1195(100); S1206(100); S1240(100); T1244(100); S1268(100); T1282(99.2); T1289(100); S1306(100); S1309(100); T1316(100); S1369(100); S1373(100); T1380(100); T1384(100); T1390(100); S1483(100); T1486(100); S1498(100); T1504(100); S1517(100); T1520(100); S1577(100); S1614(100); S1639(100); T1653(100); S1717(100); T1733(100); | 0.23 | -1.74 |
|  |  |  | T1767(100); T1782(100); T1796(100); S1835(100); T1858(100); T1861(100); S1901(100); S1922(100) | S1742(100); T1767(100); S1835(100); T1861(100); S1901(100); S1922(100) |  |  |
| Q9CYR6 | *Pgm3* | Phosphoacetylglucosamine mutase | S64(100) | Phospho [S64(100)] | 1.3 | -1.51 |
| P16015 | *Car3* | Carbonic anhydrase 3 | S9(100); S50(100); S85(100); Y114(100); T129(100); T216(99.2); S219(99); S227(100) | S48(100); S50(100); T73(100); S85(100); Y114(100); T129(100); S219(99.2) | 0.19 | 0.2 |
| Q91YE8 | *Synpo2* | Synaptopodin-2 | S543(100); S546(98.7); S596(99.2); S618(100); S629(97.8); T744(100); S767(91.5); S813(100); S895(100); S899(100) | S220(100); S543(99.2); S563(100); S566(100); S596(99); S618(100); S629(97.6); S704(100); T744(100); S813(100); S895(100); S899(100); S903(100) | -0.21 | -1.34 |
| P17751 | *Tpi1* | Triosephosphate isomerase | T121(100); S156(100); T228(100); S245(100); S273(100) | T121(100); S245(100); S254(99.4); S273(100) | 0.27 | -1.25 |
| D3YVS1 | *NA* | Smoothelin | S132(99); S313(100) | S132(98.8); S313(100) | -0.15 | -1.2 |
| G3UWY3 | *Cobl* | Protein cordon-bleu | S40(98.4); S228(100); S253(100); S265(100); S302(98.6); S310(100); S325(100); S339(100); S342(99.6); S365(100) | S40(100); S43(100); S205(100); S228(100); S253(100); S265(100); S300(95.8); S325(100); S339(100); S342(99) | 0.13 | -0.11 |
| D3Z1D3 | *3425401B19Rik* | Cardiac-enriched FHL2-interacting protein | S221(100); S252(100); S328(100); S473(100); S518(100); S677(100); S813(100); S876(98.7); S954(100); S1176(100); S1384(100) | S221(100); S252(100); S328(100); S471(99.9); S473(100); S518(100); S677(98.3); S813(100); S876(99.2); S954(100); S1347(100); S1384(100) | 0.11 | -1.17 |
| A0A0R4J1B1 | *Tnnt3* | Troponin T | S2(100); S138(100); S146(100); S182(100) | S2(100); T6(100); S146(100); S182(100) | 0.36 | -0.17 |
| A0A0G2JDV6 | *Ubap2l* | Ubiquitin associated protein 2-like | S462(94.8); S477(100); S609(100) | S477(100); S604(98.2); S609(100) | 0.12 | -1.1 |
| D3YUT2 | *Alpk3* | Alpha-protein kinase 3 | S23(100); S229(100); T545(100); S1153(100); S1165(100); S1169(100); T1184(100); S1628(100); S1638(100) | S23(100); S229(100); S361(98.9); T545(100); S1006(100); S1165(100); S1169(100); S1181(100); S1628(100); S1638(100) | -0.32 | -1.07 |
| Q8CE04 | *Cast* | Calpastatin | S82(100); S136(100) | S82(100); S136(100); T396(100) | 0.13 | -1.07 |
| Q03265 | *Atp5a1* | ATP synthase subunit alpha, mitochondrial | S53(99.2); S521(100) | S53(100); S65(100); S521(100) | 0.43 | -0.27 |
| A0A1D5RLD8 | *NA* | Glyceraldehyde-3-phosphate dehydrogenase | T81(100); T180(100); T182(99); S208(99.2); T209(98.9); S264(100); Y316(100); S319(100); S331(100) | T81(100); T182(98.9); S208(99); S264(100); S319(100); S331(100) | -0.03 | -0.99 |
| G5E8J6 | *Hrc* | Histidine rich calcium binding protein | S81(100); S104(100); S129(100); S139(100); S141(100); S150(100); S199(100); S201(100); S219(100); S228(100); S249(100); S253(100); S272(100); S305(98.5); S324(100); S332(100); Y339(100); S354(100); S376(100); S385(100); S390(100); S402(100); S421(100); S449(100); S466(100); S474(100); S483(100); S516(98.4); S520(100); S527(100); S528(100); S542(100); S565(100); S569(100); S591(100); S595(100); S640(100); S641(100) | S81(100); S104(100); S129(100); S141(99.5); S150(100); S151(100); S219(100); S228(99.2); S249(100); S253(100); S272(100); S324(100); S332(100); S354(100); S390(100); S402(98.8); T403(98.8); S421(100); S466(100); S474(100); S483(100); S516(98.1); S517(98.9); S520(100); S527(100); S528(100); S542(100); S565(100); S569(100); S591(100); S595(100); S640(100); S641(100) | 0.07 | -0.96 |
| O54724 | *Ptrf; Cavin1* | Caveolae-associated protein 1 OS=Mus musculus OX=10090 GN=Cavin1 PE=1 SV=1 | S42(99.1); S169(100); S302(99.4) | T34(99.8); S38(100); S42(100); S120(100); S169(100); S302(100) | 0 | -0.87 |
| O88990 | *Actn3* | Alpha-actinin-3 | T63(100); S115(100); S153(100); T243(100); S297(100); S320(100); S375(100); S417(100); S437(100); S439(100); S455(100); S484(100); T607(100); S610(100); S626(100); S696(100); S736(100); T750(100); S757(100); Y850(100) | T63(100); S115(100); S153(100); T184(99); T220(100); S297(100); S320(100); S375(100); S417(100); S437(100); S439(100); S601(100); T607(100); S609(100); S610(100); S626(100); T630(100); S696(100); S711(100); S722(98.4); T735(99.1); S736(98.8); T750(100); S757(100); Y850(99.2) | 0.14 | -0.33 |
| P31001 | *Des* | Desmin | S28(100); S68(100); S81(99.8); S343(100) | S25(96.8); S28(100); S48(100); S60(100); S68(100) | -0.31 | -0.96 |
| P48678 | *Lmna* | Prelamin-A/C | S22(99.2); S390(100); S392(100); S637(100) | S22(99.3); S390(100); S392(100); S637(100) | -0.05 | -0.93 |
| P70302 | *Stim1* | Stromal interaction molecule 1 | S519(100); S521(100); S575(100); S602(75); S660(100) | S512(100); S521(100); S567(100); S575(100); S660(100) | -0.23 | -0.9 |
| P70670 | *Naca* | Nascent polypeptide-associated complex subunit alpha, muscle-specific form | S257(100); S565(100); T590(100); S765(100); S822(100); S929(100); T946(98); S947(97.4); S951(98); S1039(100); S1177(100); S1208(98.7); S1285(100); S1303(100); S1400(100); S1492(100); S1579(100); S1583(100); S1715(100); S1727(98.7); S2138(100) | S249(100); S257(100); S442(100); S519(99.9); S565(100); T590(100); S822(100); S843(99.9); S929(100); S947(97.6); S951(99.9); S1039(100); S1120(100); S1174(100); S1177(100); S1285(100); S1303(100); T1398(99.9); S1400(100); T1405(96.9); S1478(100); S1489(100); S1492(99.1); S1579(100); S1583(100); S1627(98.9); S1715(100); S1727(98.8); S1744(100); T2131(96.2); S2138(100) | -0.05 | -0.9 |
| Q148W8 | *Dusp27* | Inactive dual specificity phosphatase 27 | S555(100); S562(100) | S305(100); S555(100); S966(100); T1009(91.4); S1016(99.8); T1025(100); S1029(100); S1034(100) | 0.32 | -1.01 |
| Q14BI5 | *Myom2* | Myomesin 2 | S19(100); S76(100); S78(99.1); S95(100); S106(100); S145(100); Y209(100); S239(98.8); T294(100); T296(100); Y317(100); S535(100); S555(100); T563(100); S613(100); T626(100); S737(100); S762(100); S763(99.2); T776(100); S828(100); S863(100); T1015(100); Y1024(100); Y1265(100); S1272(100); S1274(100); S1311(100); S1315(100); S1319(100); S1446(100) | S76(100); S78(100); S106(100); S145(100); S162(100); Y209(100); T294(100); T296(100); Y317(100); T445(100); S472(100); S535(100); S555(100); Y570(100); T626(100); T676(98.8); S737(100); S740(99.1); S762(100); S763(100); T776(100); S828(100); S839(100); S863(100); Y1024(100); S1046(100); S1057(100); S1102(100); Y1265(100); S1274(100); S1311(100); S1315(100); Y1400(100); S1446(100) | 0.29 | -0.4 |
| Q64347 | *Clcn1* | Chloride channel protein 1 | S682(100) | S704(100); S892(100) | 0.69 | -0.38 |
| Q80WJ7 | *Mtdh* | Protein LYRIC | S297(100); S423(100); S565(100) | S297(98.8); S565(100) | 0.05 | -0.97 |
| Q9CT10 | *Ranbp3* | Ran-binding protein 3 | S58(100); S146(100) | S58(100); T458(99.9) | 0.31 | -0.9 |
| Q9ET54 | *Palld* | Palladin | S901(100); S1141(98.8); S1146(100) | S194(100); S1129(100); S1131(100); S1141(100); S1143(100); S1146(100) | 0.31 | -0.95 |
| Q9QVP4 | *Myl7* | Myosin regulatory light chain 2 | S23(98.7) | S23(98.8) | -0.26 | -0.95 |
| A2A542 | *Cacnb1* | Voltage-dependent L-type calcium channel subunit beta-1 | S26(100); S139(100); S146(99); T158(100); T201(95.5); S454(100) | S21(90.3); S23(100); S26(100); S136(90.7); S139(100); S143(100); S145(100); S146(99); S197(96.3); S198(96.2); T201(98.4); S454(100) | 0.1 | -0.83 |
| A0A087WQ94 | *NA* | Tensin 1 | S621(99.2); S885(100); S949(100); T1174(100); S1177(100); S1194(100); S1228(97.9); S1230(97.5); S1254(100); S1282(100); S1299(100); S1366(100); S1378(100); S1385(100) | S623(98.9); S667(100); S675(95.8); S730(99); S753(100); S844(97); S885(100); S949(97.3); T1174(100); S1177(100); S1194(100); S1228(98.2); S1292(100); S1299(100); S1366(100); S1385(100) | -0.09 | -0.71 |
| A0A087WR45 | *Gm1614; Prob1* | Proline-rich basic protein 1 | S234(100); S376(100); S396(100); S398(100); S440(100); S494(100); S576(100); S772(98.7); S848(100) | S234(100); S396(100); S398(100); S440(95.1); T449(100); S451(100); S494(100); S576(100); S772(98.6); S848(100) | -0.22 | -0.75 |
| A0A087WRY3 | *Nucks1* | Nuclear ubiquitous casein and cyclin-dependent kinase substrate 1 | S19(100); S58(100); S61(100); S75(100); S79(100); S180(100); S213(100) | S19(100); S54(100); S58(100); S61(100); S75(100); S79(100); S180(100); S213(100) | 0.06 | -0.54 |
| A0A3Q4EBK4 | *NA* | Myc box-dependent-interacting protein 1 | S267(100); S273(100); T277(100); S293(100) | S267(100); S273(100); T277(100); S293(100); S303(97.9); S305(98.1) | -0.84 | -0.6 |
| A0A571BF58 | *NA* | Nebulin | S84(100); S128(100); S420(100); S513(100); Y549(100); S608(100); S667(100); Y796(100); S855(100); S1099(100); Y1325(100); S1343(100); S1587(100); S1723(100); T1753(99.1); Y1813(100); S1831(100); Y2057(100); S2075(100); Y2544(99.2); S2596(100); S2839(100); Y3030(100); S3082(100); Y3273(100); S3325(100); Y3516(100); Y3759(100); S3811(100); Y4002(100); S4020(100); T4185(98); Y4245(99.2); S4263(100); Y4730(100); S4913(100); S5015(100); T5151(100); S5445(98.9); S5480(100); Y5702(100); S5894(100); S6423(100); S6459(100); S6528(100); S6918(100); T7050(100); T7143(100); T7174(100); T7200(100); T7231(100); T7236(100) | S40(96.3); S84(100); S219(100); S362(100); S513(100); Y549(100); S584(100); S608(100); Y796(100); S831(100); S855(100); S971(100); S990(100); S1000(98.9); S1099(100); S1205(100); Y1325(100); S1723(100); T1753(99.3); Y1813(100); T1862(97.9); S2026(100); Y2057(100); S2177(100); S2420(100); Y2544(100); S2667(98); S2943(100); Y3030(100); S3186(100); Y3273(100); S3429(100); Y3516(100); Y3759(100); Y4002(100); S4020(100); Y4245(100); S4611(100); Y4730(100); T5151(100); S5445(98.8); S5645(100); Y5702(100); S5888(100); S5894(100); T5972(100); S6423(100); S6459(100); S6462(100); S6528(100); S6811(100); S6852(99) | 0.07 | -0.77 |
|  |  |  | S7257(100); T7317(100); T7346(100); S7359(100); S7374(100); S7397(100); S7405(100); S7408(100); S7419(100); S7457(100); S7460(100); T7464(98.3) | T7050(100); T7143(100); T7174(100); T7231(100); T7236(100); S7257(100); T7346(100); S7359(100); S7374(100); S7390(100); S7392(100); S7397(100); S7399(97.8); S7405(100); S7408(100); S7416(98.1); S7419(100); S7447(89.7); S7456(100); S7457(100); S7460(100); S7463(96.4); T7464(98.3); S7484(100) |  |  |
| E9QLJ0 | *Cmya5* | Cardiomyopathy-associated protein 5 | S52(100); S142(100); S155(100); S289(100); S313(98.9); S705(100); S786(100); S794(98.7); S1019(100); S1797(100); S1924(97.9); S2072(98.8); S2129(100); S2144(100); S2186(99); S2348(100); S2444(100); S2842(100); S3067(100) | S142(100); S155(100); S174(100); S199(98.4); S289(100); S705(100); S708(100); S786(99.9); S951(100); S1019(100); S1022(96.9); T1023(97.2); S1030(98.3); S1160(96.5); T1163(100); S1692(100); S1794(97.8); S1797(100); S2072(100); S2129(100); S2144(100); S2186(100); S2348(100); S2680(100); S2688(98.7); T2689(98.4); S2767(100); S2842(100); S3067(96.9); S3087(100) | 0.15 | -0.7 |
| E9QQ25 | *Speg* | Striated muscle-specific serine/threonine-protein kinase | S19(100); S316(100); S375(100); T379(100); S385(97.2); S423(100); S439(100); S463(100); S481(100); S493(100); S511(100); S518(100); T546(99.4); S554(100); S578(100); S860(100); S1177(100); S2004(100); S2019(100); S2020(100); S2042(100); S2052(100); S2114(100); S2135(100); S2171(100); S2182(98.2); S2204(96.6); S2288(100); T2303(100); S2327(100); S2347(100); S2359(100); S2361(100); S2396(100); S2413(100); S2451(100); S2461(100); S2462(99.2); S2487(99); T2488(100); S2499(100); S2562(95.2); S2777(100); T2847(100); S2936(100) | S19(100); S316(100); S375(100); T379(100); S394(100); S423(100); S439(100); T453(100); S457(100); S463(100); S490(100); S511(97.5); S518(100); S531(100); S542(100); T546(98.7); S554(100); S860(100); S865(99.9); S1177(100); S1993(98.1); S2004(100); S2019(100); S2020(100); S2042(100); S2052(100); S2099(100); S2114(100); S2135(100); S2171(98.4); S2182(100); S2204(96.4); S2288(100); T2303(100); S2327(97.1); S2361(100); S2396(100); S2413(100); S2442(97.8); S2446(95.6); S2447(95.6); S2451(100); S2461(100); S2462(99.2); S2476(100); T2488(100); S2499(100); S2503(100); S2527(96.8); S2530(96.8); S2777(100); T2847(100); S2936(100); S2944(98.4) | -0.1 | -0.82 |
| P07310 | *Ckm* | Creatine kinase M-type | S24(100); T35(100); T103(100); S128(99.3); T166(100); S178(100); T180(98.6); T313(100); T322(100); S372(100) | S24(100); T35(100); Y125(100); S128(99.3); T166(100); Y174(99.7); T313(100); T322(100); S372(100) | 0.11 | -0.69 |
| P52480 | *Pkm* | Pyruvate kinase PKM | T41(100); S57(100); S67(100); S77(100); S97(98.4); S127(99.4); Y148(100); S202(100) | T41(100); S57(100); S67(100); S77(100); Y175(100); S202(100); Y370(100) | -0.01 | -0.71 |
| P58774 | *Tpm2* | Tropomyosin beta chain | S61(100); S63(99.3); T79(100); S87(100); T108(100); Y162(100); S179(100); S206(100); T252(100); Y267(100) | S61(100); S63(99.2); T79(100); S87(100); T108(100); Y162(100); S215(99.3); T237(100); S245(100); T252(100); Y267(100); S283(99) | 0.2 | -0.48 |
| P97457 | *Mylpf* | Myosin regulatory light chain 2 | S16(99.2) | S15(100); S16(99.1); T25(96.7); T35(100) | -0.02 | -0.76 |
| Q5SX40 | *Myh1* | Myosin-1 | T64(100); T257(100); T258(100); T381(100); Y389(100); Y413(100); T415(100); T444(100); T619(100); S625(100); S649(99); T667(100); T687(100); S745(100); T761(100); T793(100); S904(100); T918(100); T986(100); T1000(100); T1026(100); T1028(100); S1044(100); S1072(99.2); S1095(100); S1135(100); S1147(100); S1165(100); T1198(100); S1206(100); S1246(100); S1264(100); T1281(100); S1291(100); S1303(100); S1309(100); T1316(100); S1334(100); Y1354(100); S1373(100); Y1382(100); T1390(100); Y1467(99.3); S1483(100); S1485(100); S1498(98.9); T1504(100); T1520(100); S1557(100); S1577(100); S1603(99.3); T1607(100); | T64(99.1); T68(100); T218(100); S225(100); T257(99.4); T258(99.2); T381(100); Y389(100); T415(100); T444(100); T619(100); S625(100); S635(100); S650(98.9); T667(100); S745(100); T761(100); T793(100); S900(100); S904(100); T944(100); T967(100); T995(100); T1000(100); T1026(100); S1044(100); S1072(100); S1095(100); S1135(100); S1147(100); S1165(100); T1195(100); S1206(100); S1229(100); S1246(100); S1248(100); S1264(100); T1289(100); S1303(100); S1309(100); T1316(100); S1334(100); Y1354(100); S1369(100); S1373(100); T1380(100); T1384(100); T1390(100); S1483(100); S1485(100); Y1495(100); T1504(100); | -0.53 | -0.55 |
|  |  |  | S1614(100); T1653(100); S1717(100); T1725(100); T1733(100); T1739(100); S1742(100); T1767(100); T1782(100); T1858(100); T1861(100); S1880(100); S1896(100); S1922(100) | S1517(100); T1520(100); S1557(100); S1577(100); S1614(100); S1639(100); T1653(100); S1717(100); T1733(100); S1742(100); T1767(100); T1861(100); S1880(100); S1896(100); S1922(100) |  |  |
| Q5XKE0 | *Mybpc2* | Myosin-binding protein C | T26(100); S40(100); S56(100); S105(97.8); S163(100); S172(100); S295(100); S404(100); S476(100); S482(100); S548(100); T583(100) | T26(100); S40(100); S105(100); S156(100); S160(100); S163(100); S172(100); S295(100); S404(100); S476(100); S482(100); S548(100); T564(100); T583(99.3); S900(98.9) | 0.66 | -0.79 |
| Q70IV5 | *Synm* | Synemin | S172(100); T430(100); T464(100); S780(100); S1044(100); S1049(100); S1075(100); S1077(100); S1502(100) | S172(100); S404(100); S405(98.5); S780(100); T811(100); S1043(97.6); S1049(100); S1075(100); S1077(100); S1087(100) | 0.06 | -0.71 |
| Q7TQ48 | *Srl* | Sarcalumenin | S228(94.5); T229(86.5); T234(95.5); T302(98.2); S304(100); S432(100); S442(100); S464(100); S491(100); T503(100); Y558(100); S592(100); Y816(100); S848(100); T873(100); S882(100) | T222(97.9); S224(97); S228(94.3); S244(100); S304(100); S317(98.1); S432(97.4); S442(100); S464(100); Y490(98.9); S491(100); T503(100); Y558(100); S569(100); S592(100); Y816(100); S848(100); S878(100); S882(100) | 0.1 | -0.52 |
| Q8CI12 | *Smtnl2* | Smoothelin-like protein 2 | S98(100); S124(99.1); S126(99.4); S131(100); S250(99.2); S265(100); T270(100); S274(100); S339(100) | S4(100); T96(100); S98(100); T122(100); S124(100); S131(100); S138(100); S250(100); S252(100); S265(100); T270(100); S274(100); S339(100) | -0.35 | -0.7 |
| Q922J3 | *Clip1* | CAP-Gly domain-containing linker protein 1 | S146(98.4); S199(100); S203(100); S311(100); S347(100) | S146(97.6); S194(100); S199(100); S203(100); S311(100); S347(100); S1317(100) | -0.15 | -0.73 |
| Q99JB8 | *Pacsin3* | Protein kinase C and casein kinase II substrate protein 3 | S319(100); S354(100); S383(100) | S276(100); S354(100); S383(100) | -0.1 | -0.51 |
| Q9DCL8 | *Ppp1r2* | Protein phosphatase inhibitor 2 | S122(100); S123(100) | S122(100); S123(100) | -0.11 | -0.62 |
| Q9JI91 | *Actn2* | Alpha-actinin-2 | T57(100); S147(100); S201(100); S291(100); S411(99.1); S431(100); S449(100); S574(100); S590(100); S594(100); S624(100); S840(100) | T57(100); S147(100); T237(100); S291(100); T308(100); S369(100); S411(99.1); T412(99); T435(100); S574(100); S590(100); S594(100); S595(97.3); T744(100); S840(100) | -0.61 | -0.72 |
| Q9JK37 | *Myoz1* | Myozenin-1 | S16(99.3); T23(100); S30(100); S57(100); S80(98.5); S82(98.8); S83(100); S118(98.5); S121(100); S134(100); S141(100); S164(100) | S15(100); S16(99.2); T23(100); S57(99.2); S80(100); S82(98.8); S83(100); S118(98.5); S121(100); S134(100); S164(100); S183(100) | 0 | -0.79 |
| Q9QXS1 | *Plec* | Plectin | S728(100); S927(100); S1443(100); T1740(100); S3448(100); T4037(100); S4389(100); S4393(100); S4396(98); S4629(100); S4633(100); S4649(100) | S728(100); S927(100); S1443(100); T1740(100); S3448(100); T4037(100); S4392(100); S4393(100); S4396(100); S4398(98.1); S4625(100); S4627(89.1); S4629(100); S4633(98.9); S4637(98); S4649(100) | -0.14 | -0.75 |
| Q9WUB3 | *Pygm* | Glycogen phosphorylase, muscle form | S26(100); Y186(100); Y204(100); S430(100); Y473(100); S524(100) | S26(100); Y186(100); Y204(100); S430(100); Y473(100); S514(100); S524(100); Y732(100); Y733(100); S748(99); S831(100) | 0.16 | -0.75 |
| Q9Z1E4 | *Gys1* | Glycogen [starch] synthase, muscle | Y44(99.1); S412(100); S641(100); S645(100); S652(100); S653(100); S657(100); S672(100); S711(98.5) | Y44(99.1); S412(100); S641(100); S645(100); S649(98.5); S653(100); S657(100); S672(100); S711(98.2); S728(99.9) | -0.06 | -0.61 |
| V9GWW6 | *NA* | Muscular LMNA-interacting protein | S61(100); S387(100); S511(93.3) | S61(100); S387(100) | -0.05 | -0.73 |
| A0A0G2JEX1 | *Nexn* | Nexilin | S80(100); S360(100); S500(100); S559(100) | S80(100); S86(100); S87(100); S345(100); S352(100); S360(100); S500(100); S559(100); T566(96.3) | 0.3 | -0.68 |
| A2AUD5 | *Tpd52l2* | Tumor protein D54 | S166(100); S175(100); S189(100) | S166(100); S171(99.1); S189(100) | -0.28 | -0.63 |
| Q8R429 | *Atp2a1* | Sarcoplasmic/endoplasmic reticulum calcium ATPase 1 | S186(100); T191(100); S229(99.2); T242(100); T441(100); T533(100); S547(100); T569(100); S581(100); S643(100) | S186(100); T191(100); T242(100); T441(100); S488(100); T533(98.6); S547(100); T554(100); T569(100); S581(100); S643(100); S693(90.9) | 0.17 | -0.68 |
| Q91VK2 | *Eef1d* | Eef1d protein | S128(100); S157(100) | T58(96.3); S128(100); T142(100); S157(100) | -0.05 | -0.64 |
| Q9ET78 | *Jph2* | Junctophilin-2 | S162(98.8); S228(100); S231(100); S234(100); T453(100); S462(100); T470(96.3); T483(100); S514(99.9); S527(100); S533(100); S613(100) | S228(100); T230(100); S231(100); S234(100); S440(99); T453(100); S462(100); T470(100); S479(95.4); T483(100); S514(100); T518(98); S520(97.9); S527(100); S533(100); S593(100); S597(100); S613(100); T621(100) | -0.05 | -0.64 |
| Q9ET80 | *Jph1* | Junctophilin-1 | S162(98.9); S165(100); S186(100); S216(100); S220(100); S238(100); S241(100); S448(100); S452(100); T461(98.1); S465(100); S469(98.1); S475(100); S480(98.8); S490(100); S496(100); S501(100); S527(100); S593(99.2) | S171(91.8); S174(99.6); T190(98.8); S216(100); S220(100); S413(100); S448(100); S452(100); T460(98.4); T461(98.2); S465(100); S468(100); S469(98.4); S475(100); S490(100); S496(100); S501(100); S532(100) | 0.23 | -0.68 |
| F8WIS9 | *Camk2a* | Calcium/calmodulin-dependent protein kinase type II subunit alpha | S333(98.9); S344(98.6) | S333(96.6); S344(100) | 0.35 | -0.64 |
| A0A0A0MQC7 | *Mapt* | Microtubule-associated protein | S188(100); S494(100); S506(100); S688(100); S696(98.6); S708(98.6) | S188(100); S491(100); S494(100); S506(100); T523(100); S529(98.9); S688(100); S692(100); S696(100) | -0.03 | -0.66 |

The red font denotes significant and differentially phosphorylated proteins (BH p-value < 0.05 and |FC| > 1.4).
